# Ultraviolet B (UVB) Photosensitivities of Tea Catechins and the Relevant Chemical Conversions

**DOI:** 10.3390/molecules21101345

**Published:** 2016-10-10

**Authors:** Meng Shi, Ying Nie, Xin-Qiang Zheng, Jian-Liang Lu, Yue-Rong Liang, Jian-Hui Ye

**Affiliations:** Tea Research Institute, Zhejiang University, Hangzhou 310058, China; 21316161@zju.edu.cn (M.S.); 21416073@zju.edu.cn (Y.N.); xqzheng@zju.edu.cn (X.-Q.Z.); jllu@zju.edu.cn (J.-L.L.); yrliang@zju.edu.cn (Y.-R.L.)

**Keywords:** photostability, catechins, photoisomerization, photolysis, photoproducts

## Abstract

Ultraviolet B (UVB) photosensitivities of eight catechins were screened. In both water and ethanol, epicatechin (EC, 575 μM) and catechin (C, 575 μM) exhibited low photostabilities under 6 h UVB radiation with the generation of yellow photoproducts, while other catechins (epigallocatechin gallate, epigallocatechin, epicatechin gallate, gallocatechingallate, gallocatechin, catechin gallate) were relatively UVB-insensitive. Photoisomerization and photolysis were two important UVB-induced reactions to EC whereas photolysis was the dominant reaction for C. The influencing factors of time (2–10 h), solvent (water, ethanol) and substrate concentration (71.875–1150 μM) on UVB-induced chemical conversions of EC and C were investigated, and eight photoproducts were identified through ultra performance liquid chromatography-diode array detection-tandem mass spectrometry (UPLC-DAD-MS/MS) and ^1^H nuclear magnetic resonance (^1^H-NMR analysis). Photolysis reaction involved two pathways, including radical reaction and photo-induced electron transfer reaction. The 2,2-diphenylpicrylhydrazyl (DPPH) scavenging abilities of eight catechins did not change upon 6 h UVB irradiation. EC and C are photosensitive catechins among eight catechins causing deep color.

## 1. Introduction

Tea catechins are the major components of the tea polyphenols present in tea leaves, which have been associated with many health benefits and functionalities such as anti-radical, anti-bacterial, anticarcinogenic and anti-aging activities [1,2]. They are mainly composed of (−)-epigallocatechin gallate (EGCg), (−)-epigallocatechin (EGC), (−)-epicatechin gallate (ECg), (−)-epicatechin(EC), and their geometric epimers (−)-gallocatechingallate (GCg), (−)-gallocatechin (GC), (−)-catechin gallate (Cg), and (−)-catechin (C). In the past decades, the protective effects of green tea or tea extract against Ultraviolet (UV)-induced skin damage and skin aging have been reported [3,4,5], leading to the growing interest in utilization of green tea or tea extract for cosmetics and skin creams [6,7,8]. However, tea catechins or tea extracts inevitably turn brown during storage, which limits their application not only in the cosmetic industry but also in the food industry due to the undesirable dark color. The bioactivity changes of catechins upon browning draw great interest [9,10].

Illumination, temperature and pH value are important influential factors for non-enzymatic browning of catechin-containing products [11]. The stabilities of catechins at different temperatures and pH values have been excessively studied [12,13,14,15]. Epimerization, hydrolysis and oxidation/condensation reactions are three important reactions accounting for instability or degradation of catechins with the generation of epimer, gallic acid (GA) and non-gallated catechin moiety, as well as dark polymeric compounds [12,13,14,15]. However, these may not represent the photo-induced chemical reactions of tea catechins that are driven by an entirely different energy. Illumination is a nonnegligible environmental condition for storage, thus the photostabilities of tea catechins deserve a systematic investigation in order to resolve the browning problem of tea catechins caused by illumination.

Ultraviolet B (UVB) is electromagnetic radiation with a wavelength ranging from 320 nm to 290 nm, and is regarded as the most destructive natural radiation on account of its energy, which is high enough to cause many photochemical reactions [16]. UVB radiation has been used to investigate the photosensitivity of natural polyphenols due to the advantages of its constant and adjustable intensity, easy control and simplicity compared with solar radiation [17,18,19]. Various phenolic compounds, such as apigenin, quercetin, resveratrol, EGCg, C and EC have been reported to be susceptible to photo-oxidation in the presence or absence of exogenous photosensitizers/free radical initiators [17,18,19,20,21,22]. Severe photodegradation of EGCg in topical cream was reported with the degradation percentage being ~70% [9,23], and 83% of C (950 μM, methanol) was lost after 3 h of ultraviolet C (UVC) exposure [24]. Phenoxyl radicals were produced from the catechol or resorcinol rings of C by photo-oxidation, leading to further chemical evolution [25]. For the eight catechins, substituent groups may impact their chemical properties, resulting in various responses to UVB radiation and different contributions to browning process. However, relevant information on the photosensitivities of the eight catechins is insufficient.

In the present work, the photostabilities of eight catechins under UVB radiation were investigated, and the UVB-susceptible catechins were selected for further studies in terms of the effects of solvent, radiation time and substrate concentration. Photoproducts were identified by ultra performance liquid chromatography-diode array detection-tandem mass spectrometry (UPLC-DAD-MS/MS) and ^1^H nuclear magnetic resonance (NMR), and the reaction mechanisms of UVB-induced transformations were discussed. The impact of UVB radiation on the antioxidant activities of the eight catechins was evaluated by 2,2-diphenylpicrylhydrazyl (DPPH) assay.

## 2. Results and Discussion

### 2.1. The Photostabilities of Catechins under UVB Radiation

One molecule of catechin converts to one molecule of its epimer through an epimerization reaction [26], and one molecule of gallated catechins produces one molecule of GA via hydrolysis of the ester bond [27]. The percentage of conversion of catechins due to epimerization and hydrolysis reactions was calculated by the equation in Table 1. The percentage of catechin loss due to other non-identified reactions was calculated by subtracting the percentage of epimerization and the percentage of hydrolysis from 100%, and was termed other percentage degradation for representation. Table 1 shows the photostabilities of individual catechins in both water and ethanol. After 6 h of UVB radiation, no GA was detected in any of the catechin samples, indicating that no hydrolysis reaction occurred under UVB radiation. Therefore the GA data was not presented in the following studies. In Table 1, GC, EGC, GCg, EGCg, Cg and ECg were insensitive to UVB irradiation with percentage maintained of 90.7%–99.6%, whereas only 58.0%–80.3% of EC and C were preserved after 6 h UVB radiation. A decrease below 10% is considered as insensitivity to UVB. Hence, EC and C both in water and ethanol showed high photosensitivities to UVB, which was also reflected by their color change from colorless to yellow under UVB radiation (Table 1). Epimers were only detected in the aqueous and ethanol solutions of EC and C but not the other six catechins. For EC, 31.1% and 20.0% converted to its epimer C in water and ethanol respectively, compared with 2.6% and 5.2% of C converting to EC accordingly, which suggests that EC and C undergo isomerization under UVB radiation and that the epi-type catechin is favorable for photoisomerization. Forest et al. [28] also reported that photoisomerization occurred to C with the generation of unknown yellow compounds as side products. The sum of the percentage of epimerization and the percentage maintained of EC and C in both water and ethanol were 64.2%–90.4% (Table 1), indicating that other reactions played an important part in the UVB-induced chemical transformations of EC and C in addition to photoisomerization. Specifically, the unidentified reactions respectively cost 9.6% and 22.0% of EC, and 17.1% and 35.8% of C in water and ethanol, suggesting that C was more susceptible to the unknown UVB-induced reactions. EC and C showed the highest photosensitivities among the eight catechins and were therefore selected for further study.

In our study, EC and C, the basic unit of flavan-3-ols, exhibited high susceptibility to UVB radiation whereas EGC, GC, EGCg, GCg, ECg and Cg were UVB-insensitive, indicating that the presence of gallated and pyrogallol moieties hindered the UVB-induced chemical transformations. This is in accordance with the previous report showing that epimerization reaction was closely related with chemical structure of flavan-3-ols [29]. Dobashi et al. [17] demonstrated that EC and C showed higher photo-antioxidant activity than EGC whereas no photo-antioxidant activity of gallated catechins was observed. Hence, a substituent group has a decisive influence on the photosensitivity of flavan-3-ols. In addition, a clear solvation effect on the photoisomerization of EC and C was observed (Table 1). An aqueous solution was favorable to photoisomerization. It was reported that UVB-induced epimerization of EC to C was via an intermediate quinone methide through ionization [20]. Thus, higher polarity of water facilitated the ionization process, increasing the generation of intermediate quinone methide compared with ethanol medium. This explained why more EC converted to C in water than ethanol. Solvation effect on epimerization reaction of catechins was also reported in other studies [29,30]. Solvation effect on the unidentified reaction was discussed in a later section.

### 2.2. The Chemical Transformations of EC and C with UV B Radiation Time

**Figure 1:**
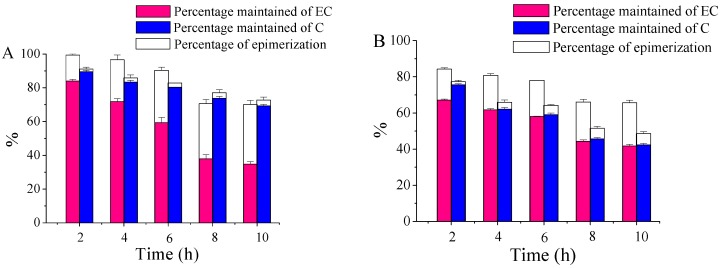
The changes in percentage of epimerization and the percentage maintained of EC and C with UVB radiation time: (**A**) water, 575 μM; (**B**) ethanol, 575 μM.

There are different types of reactions occurring to EC and C under UVB radiation, which might play differently with radiation time. Figure 1 shows the changes in percentages of epimerization and percentages maintained of EC and C over time. The corresponding percentages of degradation due to other reactions can be obtained by subtracting the percentage of epimerization and the percentage maintained from 100%. Upon 2 h of UVB irradiation, 15.4% and 0.5% of EC in water underwent epimerization and degradation reactions respectively, compared with 17.2% and 15.7% of EC in ethanol (Figure 1). Upon 2 h of UVB radiation, the percentage of epimerization and percentage of degradation of C were 1.7% and 8.8% in water, and 1.7% and 22.7% in ethanol (Figure 1). These results indicate that epimerization was the dominant reaction for EC in both water and ethanol for the first 2 h of UVB radiation, and other degradation reactions were the principal reactions to C under the same conditions. As radiation time increased, the percentage of epimerization of EC in water increased rapidly after the first 8 h and then maintained stable at ~35.4% from 8 h to 10 h, while the percentage of degradation of EC slowly increased from 0.5% to 9.6% after the first 6 h and then rapidly increased from 9.6% to 29.9% in the subsequent 4 h (Figure 1A). This suggests an antagonistic relationship between percentage of epimerization and percentage of degradation of EC in water, which is possibly due to the competition for UVB energy between epimerization and degradation reactions. The percentages of degradation of EC and C in ethanol gradually increased with radiation time, and were much higher than those in water. Thus, ethanol medium exacerbated the photo-degradation of EC and C. The 6 h point was selected for the following studies on account of the high percentage of conversion in both epimerization and degradation reactions.

### 2.3. The Effect of Substrate Concentrations of EC and C

**Figure 2:**
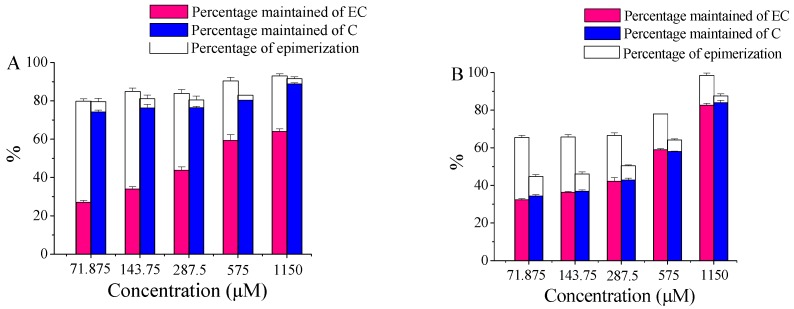
The effect of substrate concentration on the percentage of epimerization and percentage maintained of EC and C under 6 h UVB radiation: (**A**) water and (**B**) ethanol.

Figure 2 shows the effect of substrate concentration on percentage of epimerization and percentage maintained of EC and C in water and ethanol. As substrate concentration increased from 71.875 to 1150 μM, the percentage of epimerization and percentage of degradation of EC and C in both water and ethanol decreased and resulted in an elevated percentage of maintained EC and C at high substrate concentration. This indicates that high substrate concentration suppressed the UVB-induced chemical transformations of EC and C. From Figure 2, we can see that the solvent exerted a great influence on the chemical conversions of EC and C under UVB radiation. At the substrate concentration of 71.875–1150 μM, the percentages of epimerization of EC were 29.0%–52.7% for water and 5.8%–33.2% for ethanol, while the percentages of degradation of EC in water and ethanol were 7%–20.2% and 1.5%–34.5% respectively. Hence, in contrast to water, ethanol medium suppressed the photoisomerization of EC but increased the percentages of degradation of EC at low substrate concentration (71.875 μM to 575 μM). The percentages of epimerization of C in water and ethanol were less than 10.4%, while the percentages of degradation of C were 8.4%–20.4% for water and 12.4%–55.3% for ethanol, hence other reaction was the dominating reaction to C upon UVB irradiation (Figure 2). This was consistent with previous result showing that epi-structure is favorable for photoisomerization of catechin.

### 2.4. Structural Characterization of Photoproducts

**Figure 3:**
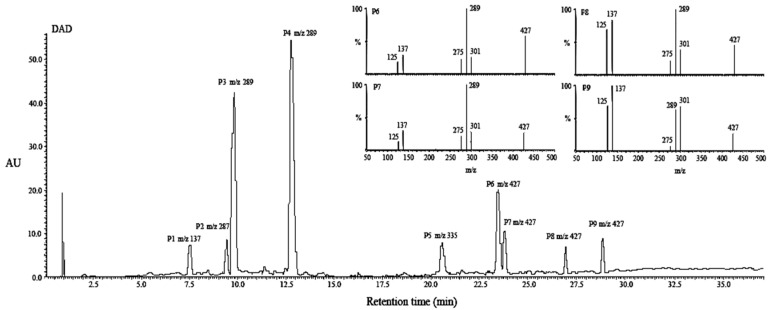
The ultra performance liquid chromatography (UPLC) and mass spectrometry (MS) information of UVB irradiated EC in ethanol after concentration. Inset P6-9: the MS/MS spectra of photoproducts assigned to P6-9.

**Figure 4:**
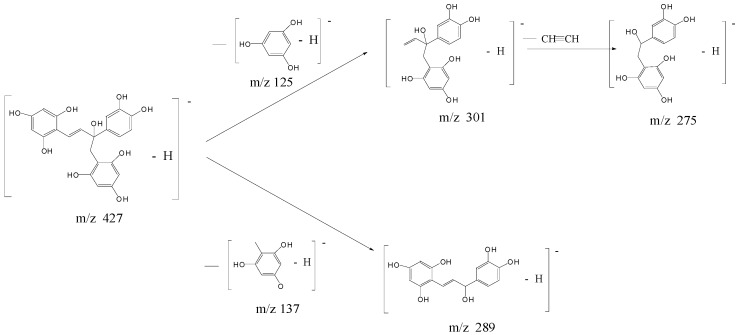
Proposed fragmentation pathway of isomeric ions at [M − H]^−^^1^ *m*/*z* 427.

Based on UPLC-DAD-MS analysis, the UVB irradiated aqueous and ethanol solutions of EC and C had the same types of photoproducts. The UPLC chromatogram and MS information of concentrated EC sample (287.5 μM in ethanol, 6 h UVB radiation) has been presented in Figure 3 as an example. Except for the typical peaks corresponding to EC (P4, [M − H]^−1^ *m*/*z* 289) and its epimer C (P3, [M − H]^−1^ *m*/*z* 289), seven new peaks for P1, P2, P5–9 were detected with [M − H]^−1^ *m*/*z* at 137, 287, 335, 427, 427, 427, 427 (in order), suggesting that new photoproducts were generated under UVB radiation due to the unidentified reactions. The photolytic cleavage of flavan-3-ols (fisetinidol and C) in methanol occurred at the ether linkage C-O of the heterocyclic ring with production of the corresponding diradicals. These diradicals underwent two pathways: radical reaction and photo-induced electron transfer reaction, which respectively led to the generation of *ortho*-quinone methides and 1,3-diarylpropan-2-ols with an addition reaction of methanol to the diradical [18]. In the case of EC (molecular weight abbreviated M_r_, 290 Da) as reactant and ethanol as medium (M_r_ 46 Da), theoretically the molecular weights of *ortho*-quinone methide-like and 1,3-diarylpropan-2-ols-like products should be 138 Da and 336 Da, which was observed in Figure 3 corresponding to P1 and P5 with [M − H]^−1^ *m*/*z* 137 and *m*/*z* 335 respectively. Compound P2 at [M − H]^−1^ *m*/*z* 287 was considered as quinone on account of the typical transformation from phenol to quinone with a loss of 2H [31], and this was affirmed later by its UV adsorption spectrum. New types of photoproducts P6-9 were found with [M − H]^−1^ *m*/*z* 427 in Figure 3. Coincidently, the M_r_ of new compounds (428 Da) was the sum of 138 and 290 Da, suggesting that these four compounds are possibly the coupling products of the biradical of EC (M_r_ 290 Da) and the radical precursor of *ortho*-quinone methide (M_r_ 138 Da). Hence, a daughter ion scan MS experiment with a mass of parent ion set at 427 *m*/*z* was carried out to further investigate the molecular structures of Compounds P6-9, and their MS/MS spectra are shown in the inset of Figure 3. Obviously, Compounds P6-9 were isomers on account of the same [M − H]^−1^ *m*/*z* 427 and similar MS/MS spectra. Based on our speculation that Compounds P6-9 might be formed from addition of *ortho*-quinone methide-like compound to the biradical of EC, the proposed fragmentation pathway of isomers P6-9 is shown in Figure 4, which was in an agreement with the MS/MS spectrum. The presence of one double bond and one chiral carbon in the molecule is the reason for the existence of four isomers.

**Figure 5:**
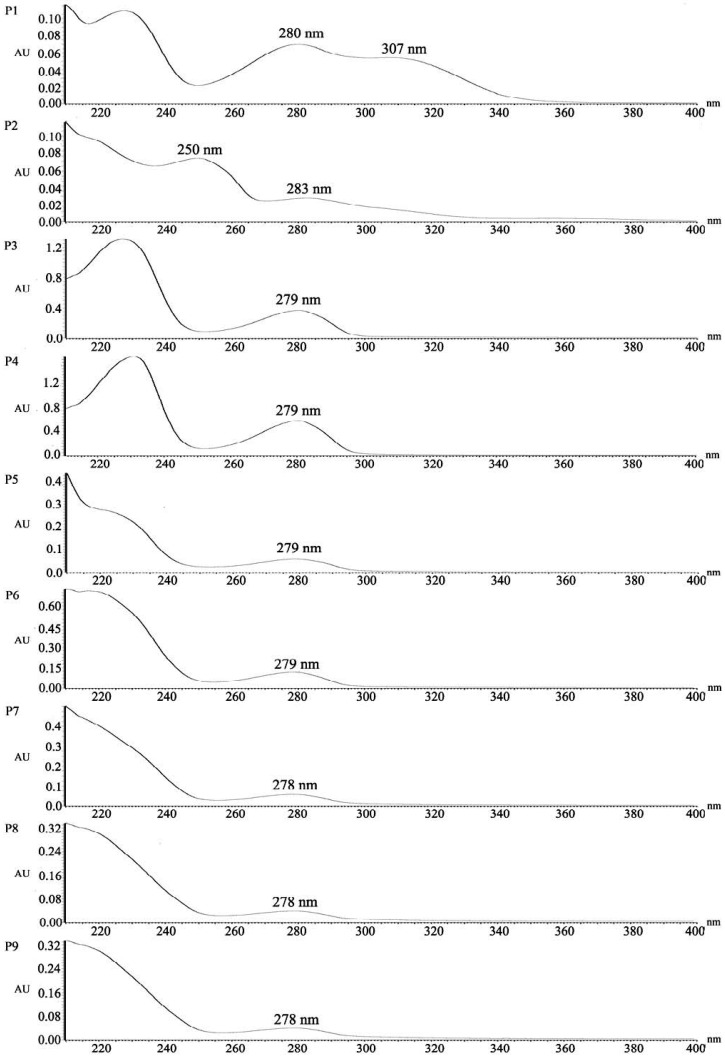
The ultraviolet (UV) spectra of Compounds P1-9.

The UV-visible spectra (190–400 nm) of all compounds are shown in Figure 5. The UV-visible spectra of Compounds P1 and P2 exhibited two strong absorption peaks at 280 nm/307 nm and 250 nm/283 nm respectively, which was distinctly different from the maximum absorbance at ~280 nm of Compounds P3-9 due to aromatic ring. The *para*-Quinone methides have typical UV absorbance at 300 nm and benzoquinone has strong absorbance at 244 nm [32,33]. The UV absorption spectra also confirmed that Compounds P1 and P2 belonged to quinone methides and quinone respectively, due to the possession of characteristic absorption bands near 300 nm and 244 nm. The less than 10 nm difference might be attributable to the substitution of hydroxyl groups on aromatic ring leading to a shift of λ_max_ value to longer wavelength (bathochromic effect) because of electron donating effect of hydroxyls. In addition, *ortho*-Quinone methides and quinone are responsible for the yellow color of UVB irradiated EC sample, since quinonoid compounds are often yellow to red in color [34].

The ^1^H-NMR spectra of EC and its UVB irradiated reaction mixture with higher percentages of conversion were studied to resolve the characteristic ^1^H frequency bands of new photoproducts from EC for further testifying the chemical structures of photoproducts. Since nine compounds are present in the UVB irradiated reaction mixtures including four isomeric products and several products at a low level, it is not practical to isolate each photoproduct. The NMR spectra data of EC is as follows: ^1^H-NMR of EC (600 MHz, Acetone-d6) δ ppm: 8.10 (s, H, H-3’), 7.94 (s, H, OH-5), 7.79 (s, H, OH-4’), 7.73 (s, H, OH-7), 7.05 (d, 1H, H-2’, *J*_2’,6’_ = 1.7 Hz), 6.83 (dd, 1H, H-6’, *J*_6’,2’_ = 1.7 Hz, *J*_6’,5’_ = 8.3 Hz), 6.78 (d, 1H, H-5’, *J*_5’,6’_ = 8.3 Hz), 6.02 (d, 1H, H-6, *J*_6,8_ = 2.3 Hz), 5.91 (d, 1H, H-8, *J*_8,6_ = 2.3 H), 4.88 (br, s, 1H, H-2), 4.21 (m, 1H, H-3), 3.58 (d, 1H, OH-3, *J*_OH-3,3_ = 5.5 Hz), 2.86 (dd, 1H, H-4a, *J*_4a,4b_ = 16.6 Hz, *J*_4a,3_ = 4.7 Hz), 2.74 (dd, 1H, H-4b, *J*_4b,4a_ = 16.6 Hz, *J*_4b,3_ = 3.2 Hz). The ^1^H-NMR data of EC was consistent with reported results [15,35]. After UVB radiation, new ^1^H frequency bands were found in the ^1^H-NMR spectra of reaction mixture from EC in addition to the original bands of EC, including 1.12 ppm (t, *J* = 6.8 Hz) corresponding to methyl proton of characteristic branch -O-CH_2_-C**H**_3_ in new Compound 5, 2.91 ppm (dd, *J* = 5.4 Hz, *J* = 16.1 Hz) corresponding to methylene proton of characteristic branch -O-C**H**_2_-CH_3_ in new Compound P5, 6.89 ppm (d) and 6.85 ppm (d) corresponding to protons on the carbon-carbon double bond in new Compounds P6-9. This indicated the presence of speculated functional group or structure in the UVB irradiated reaction mixture of EC.

### 2.5. Proposed Reaction Mechanisms

**Figure 6:**
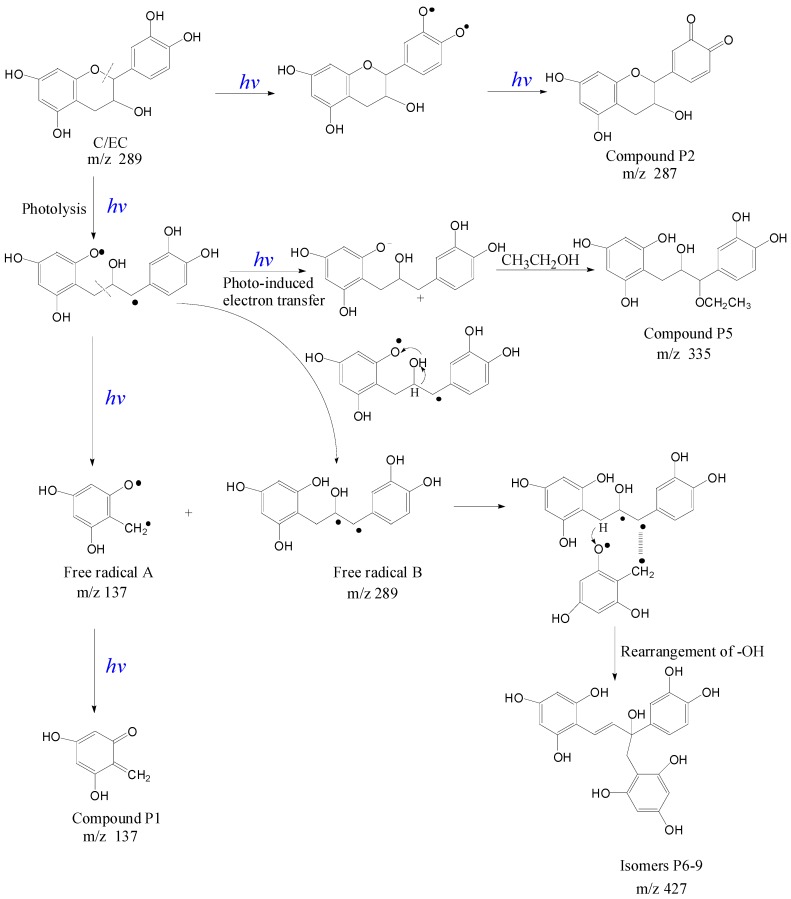
Proposed photolytic reaction mechanism of EC/C.

The photolytic reaction mechanism of EC/C is given in Figure 6, referring to the reported two photolysis pathways of flavan-3-ols [18]. Upon UVB irradiation, EC/C molecule was excited at two positions: -OH bond and heterocyclic ring. The excitation of -OH bond led to the generation of quinone compound P2 at [M − H]^−1^ *m*/*z* 287, while the heterocyclic ring of EC/C was preferentially opened via photolytic cleavage at the ether linkage C-O with low bond dissociation energies, which resulted in the generation of free radicals A and B at *m*/*z* 137 and 289. Free radical reaction led to the generation of quinone methides such as Compound P1 at *m*/*z* 137 and four isomers P6-9 at *m*/*z* 427 through grafting reaction of free radical A *m*/*z* 137 onto free radical B *m*/*z* 289 after intramolecular rearrangement of H and -OH (Figure 6). The phloroglucinol grafted derivatives of EC and C were also photosynthesized by Wilhelm-Mouton et al. [36] via photolytic cleavage of the ether bond on the heterocyclic ring of flavan-3-ols. Besides, neutral radicals can also be ionized in a polar solvent due to photo-induced electron transfer reaction [37], which is the other pathway of photolysis in competition with free radical reaction. The photo-induced electron transfer reaction product at *m*/*z* 335 was obtained in our study with an addition of -OCH_2_CH_3_ from ethanol, indicating the occurrence of electron transfer reaction under UVB radiation. Thus, photolysis reaction was responsible for the percentages of EC and C loss previously termed percentages of degradation. In the present study, two reaction pathways of radical and photo-induced electron transfer reactions synchronously occurred to UVB irradiated EC and C with the generation of three types of photoproducts, which complemented the reported conclusion [18].

### 2.6. Antioxidant Activities

The antioxidant activities of individual catechins (575 μM, ethanol) determined by DPPH assay were shown in Table 2. EGCg had the highest DPPH scavenging abilities; 2064 ± 29 μM trolox, followed by GCg, ECg, Cg, EGC, GC, EC and C in that order, which indicates that gallated catechins possessed higher antioxidant capacities than non-gallated catechins and epi-type catechin was more antioxidant active than the corresponding non-epi type at the same molality. This result was consistent with the antioxidant activity rank of different catechins reported by Lee et al. [38]. Upon 6 h of UVB irradiation, no significant difference in DPPH scavenging ability was observed for the eight catechins. This was in line with previous result that no obvious photo-degradation was observed for catechins except EC and C under 6 h UVB radiation. For EC and C, photoisomerization and photolysis reaction may not significantly influence their antioxidant activities in the short-term but could be an important cause of browning deterioration of catechins-containing products in color. Besides, a yellowish color change was also observed in the aqueous solutions of EC and C after 15-day-storage under laboratory illumination (Supplementary Materials Figure S1), indicating that UVB radiation is not a necessary condition for photo-degradation of EC and C. Nevertheless, the photoproducts of EC and C under laboratory illumination need identification. The photosensitivities of EC and C provide a new aspect for solving the browning problem of catechin-containing products via diminishing color deterioration originating from EC and C.

## 3. Materials and Methods

### 3.1. Chemicals

The individual catechins (−)-epigallocatechin gallate (EGCg, ≥98%), (−)-epigallocatechin (EGC, ≥98%), (−)-epicatechin gallate (ECg, ≥98%), (−)-epicatechin (EC, ≥98%), (−)-gallocatechingallate (GCg, ≥98%), (−)-gallocatechin (GC, ≥98%), (−)-catechin gallate (Cg, ≥98%), (−)-catechin (C, ≥98%), and gallic acid (GA, ≥98%) were purchased from Aladdin Industrial Corporation (Shanghai, China). The DPPH was purchased from Tokyo Chemical Industry Co., Ltd. (Tokyo, Japan). The (±)-6-Hydroxy-2,5,7,8-tetramethylchromane-2-carboxylic acid (Trolox) was purchased from Sigma-Aldrich (Shanghai, China). Chemical purity grade ethanol was purchased from Sinopharm Chemical Reagent Co., Ltd. (Beijing, China). Acetic acid glacial (TEDIA company, Fairfield, OH, USA), methanol and acetonitrile (Avantor performance materials, Inc., Center Valley, PA, USA) were of HPLC (High performance liquid chromatography) grade. The Milli-Q water was prepared by an EASYPure II UV UltraPure Water System (Barnstead International, Dubuque, IA, USA).

### 3.2. Photosensitivities of Eight Catechins to UVB

The solutions of individual catechins were freshly prepared at 575 μM with water and ethanol respectively. Two milliliters of each catechin solution was placed in a 2 mL polypropylene centrifuge tube which was exposed to UVB for 6 h with a fully opening lid, and was then stored at −20 °C to terminate reactions. The intensity of UVB was achieved at 100 μW·cm^−2^ by placing samples under six UV lamps (SPECTRONICS BLE-1T158 Tube 15 watt, main output at 312 nm) at a distance of 45 cm, which was close to the UVB intensity in the sunlight. All samples were turned back to room temperature, and made up to 2 mL prior to HPLC analysis.

In a pretest, the potential UVB absorbing effect of polypropylene tube wall on the UVB-induced chemical conversion of catechins (575 μM, ethanol) was evaluated by comparing with UVB-transmissive Q-cuvette. The result showed that the photo-induced conversion rates of EC and C in polypropylene tubes slightly decreased by ~8% compared with Q-cuvette, while no significant difference was observed for other catechins. The polypropylene centrifuge tubes were used in the following studies considering operational convenience and practical use.

### 3.3. Effect of UVB Radiation Time on Conversions of EC and C

According to the recovery of catechins in Section 3.2, EC and C were UVB-susceptible and selected for further investigation. The solutions of EC and C were prepared at 575 μM with water and ethanol respectively. Two milliliters of EC and C solutions were exposed to UVB for 2 h, 4 h, 6 h, 8 h and 10 h, respectively. All samples were treated as above and analyzed by HPLC.

### 3.4. Effect of Substrate Concentration on Conversions of EC and C

The solutions of EC and C were respectively prepared at 71.875, 143.75, 287.5, 575 and 1150 μM by water and ethanol. Two milliliters of C and EC solutions at various concentrations were exposed to UVB for 6 h. All samples were treated as described in Section 3.2 and analyzed by HPLC.

### 3.5. HPLC Analysis

All samples were centrifuged at 12,000 rpm for 10 min before HPLC analysis. The concentrations of catechins and GA were analyzed by HPLC according to a previous paper [39]. The HPLC conditions were: injection volume 10 μL, Agilent TC-C18 column (4.6 mm × 250 mm, Agilent Technologies, Santa Clara, CA, USA), column temperature 32 °C, mobile phase A = acetonitrile/acetic acid/water (6:1:193, v), mobile phase B = acetonitrile/acetic acid/water (60:1:139, v), linear gradient elution: from 80% (v) A and 20% (v) B to 35% (v) A and 65% (v) B for the first 35 min and then 80% (v) A and 20% (v) B until 40 min, flow rate 1 mL·min^−1^, Shimadzu SPD ultraviolet detector at 280 nm. Catechins were quantified according to external standard calibration by comparing with the corresponding authentic standards.

### 3.6. Identification of the Photoproducts of EC and C

Ninety milliliters of the aqueous and ethanol solutions of EC and C (287.5 μM) were respectively exposed to UVB for 6 h as described in Section 3.2. The reaction mixtures of EC and C were submitted to rotary evaporator, concentrated to 10 mL at 40 °C under vacuum and sampled for UPLC-DAD-MS/MS analysis and the left concentrate was freeze dried for ^1^H-NMR measurement.

#### 3.6.1. UPLC-DAD-MS/MS Analysis

UPLC-DAD-MS/MS (Waters Corporation, Milford, DE, USA) was employed to provide the MS information of relevant photoproducts. The UPLC conditions were: Acquity UPLC HSST3 column (2.1 mm × 150 mm, 1.8 μm), column temperature 35 °C, injection volume 5 μL, mobile phase A = 0.1% formic acid + 99.9% water (*v*/*v*), mobile phase B = 0.1% formic acid + 99.9% acetonitrile (*v*/*v*), linear gradient elution: from 99.9% (v) A/0.1% (v) B to 10% (v) A/90% (v) B for 38 min, flow rate 0.3 mL·min^−1^. An electrospray ionization (ESI) technique in a negative ion mode was employed for MS analysis. The ion source conditions were set as follows: capillary voltage 3000 V, cone voltage 30 V, extractor 3.0 V and RF lens 0.2 V, ion source temperature 150 °C, desolvation gas nitrogen at a flow rate of 800 L·h^−1^ and temperature at 300 °C. Full scan ranging from 100 to 1000 atomic mass unit (amu) were recorded. Argon was used as the collision gas. The collision energy was 20 eV. Triple quadrupole was set-up to daughter ion scan experiment, and the mass of parent ion was set at 427 *m*/*z*. UV-visible spectrum of reaction products was recorded in the 190–400 nm range by a photodiode detector.

#### 3.6.2. ^1^H Nuclear Magnetic Resonance (NMR) Measurement

^1^H-NMR spectra were obtained from ~5 mg of EC and the corresponding reaction mixture suspended in 0.8 mL acetone-d 6. The spectra were recorded on an Agilent 600 MHz DD 2 with a 5 mm One NMR probe (Agilent Technologies, Santa Clara, CA, USA). Integration of the spectra was performed with Mnova NMR software (version 6.1.0, Mestrelab Research S. L., Santiago de Compostela, Spain).

### 3.7. Antioxidant Activity Measurement

Antioxidant activity was evaluated by scavenging activity of DPPH free radical according to the modified method [40]. One hundred and eighty microliters of DPPH solution (0.4 mM, ethanol) and 20 μL of individual catechin solution (575 μM, ethanol) with and without 6 h UVB radiation were loaded onto a microplate, and then the mixture was incubated in dark at 30 °C for 30 min and measured by Epoch Microplate Spectrophotometer (BioTek Instruments Inc., Winooski, VT, USA) at 517 nm. Antioxidant capacity was expressed as μM trolox equivalents, using the linear calibration curve of trolox (100–1250 μM).

### 3.8. Data Analysis

All analyses were carried out in triplicate. The mean values of the triplicate analysis ±SD are presented.

## 4. Conclusions

Eight catechins showed different photosensitivities to UVB. EC and C were susceptible under UVB radiation while other six catechins were insensitive. Photoisomerization and photolysis were the main reactions in the photo-induced chemical transformations of EC and C, which were influenced by radiation time, solvent and substrate concentration. Two reaction pathways including radical reaction and photo-induced electron transfer reaction were involved in the photolysis reaction of EC and C. No significant change was observed in the antioxidant activities of eight catechins upon 6 h UVB irradiation.

## Figures and Tables

**Table 1:** The photostabilities of eight catechins under ultraviolet B (UVB) radiation (μM) ^1^.

Catechins ^2^	Molecular Structures	After UV Radiation ^3^	Compositions	Water ^4^	Ethanol ^4^
EC	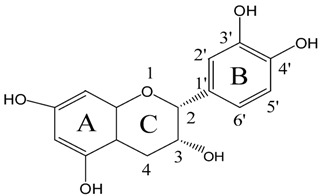	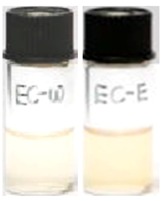	EC	341.0 ± 6.3 (59.3 ± 3.1%)	333.7 ± 1.1 (58.0 ± 0.2%)
			Epimer	178.5 ± 3.6 (31.1 ± 1.2%)	115.0 ± 0.7 (20.0 ± 0.1%)
C	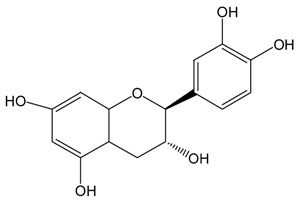	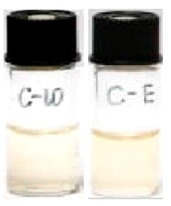	C	461.5 ± 4.5 (80.3 ± 0.0%)	339.2 ± 3.4 (59.0 ± 0.6%)
			Epimer	14.9 ± 0.1 (2.6 ± 0.0%)	30.0 ± 0.5 (5.2 ± 0.1%)
EGC	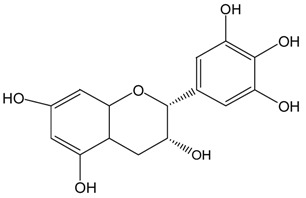	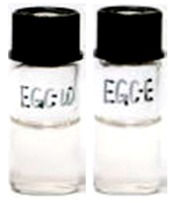	EGC	531.6 ± 9.4 (92.5 ± 1.6%)	521.6 ± 7.1 (90.7 ± 1.2%)
			Epimer	UD	UD
GC	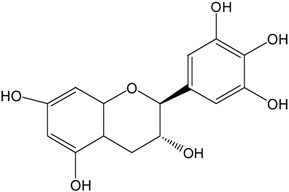	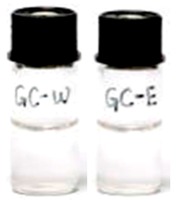	GC	557.5 ± 3.3 (97.0 ± 3.2%)	546.7 ± 1.8 (95.1 ± 0.3%)
			Epimer	UD	UD
ECg	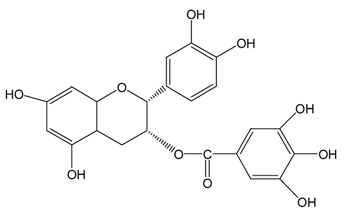	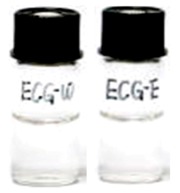	ECg	567.7 ± 1.2 (98.7 ± 0.2%)	544.1 ± 4.1 (94.6 ± 0.7%)
			Epimer	UD	UD
Cg	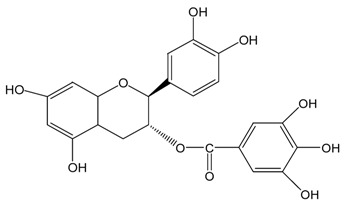	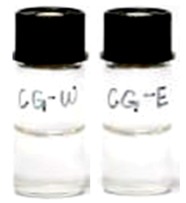	Cg	569.8 ± 3.0 (99.1 ± 0.5%)	536.9 ± 3.8 (93.4 ± 0.7%)
			Epimer	UD	UD
EGCg	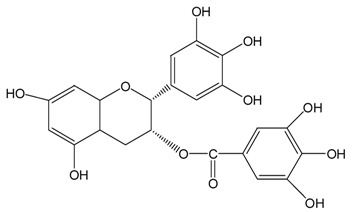	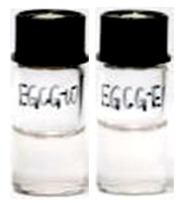	EGCg	572.4 ± 2.6 (99.6 ± 0.5%)	525.3 ± 0.7 (91.4 ± 0.1%)
			Epimer	UD	UD
GCg	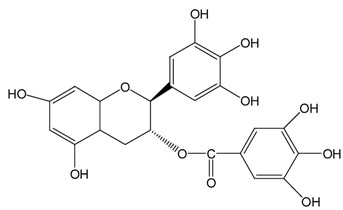	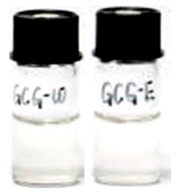	GCg	566.2 ± 0.0 (98.5 ± 0.0%)	524.0 ± 2.8 (91.1 ± 0.5%)
			Epimer	UD	UD

^1^ The solutions of individual catechins were prepared at 575 μM with water and ethanol respectively; ^2^ EC-C, EGC-GC, ECg-Cg, EGCg-GCg are geometric isomers; ^3^ Pictures of the water and ethanol solutions of individual catechins after 6 h UVB radiation: W-water; E-ethanol; ^4^ Data in round blankets were the maintained percentage of catechins. Data in square blankets were the percentages of epimerization of individual catechins = concentration of epimer (μM)/575 μM × 100%. EC, (−)-epicatechin; C, (−)-catechin; EGC, (−)-epigallocatechin; GC, (−)-gallocatechin; ECg, (−)-epicatechin gallate; Cg, (−)-catechin gallate; EGCg, (−)-epigallocatechin gallate; GCg, (−)-gallocatechin gallate; UD, undetectable.

**Table 2:** The 2,2-diphenylpicrylhydrazyl (DPPH) scavenging abilities of individual catechins before and after UVB radiation (μM trolox) ^1^.

Treatment	EC	C	EGC	GC	ECg	Cg	EGCg	GCg
Before UVB radiation	1219 ± 49	967 ± 48	1376 ± 27	1285 ± 7	1836 ± 28	1736 ± 43	2064 ± 29	1996 ± 53
After UVB radiation	1136 ± 33	921 ± 30	1363 ± 12	1274 ± 30	1808 ± 31	1745 ± 23	2064 ± 22	2026 ± 21

^1^ The ethanol solutions of individual catechins (575 μM) were UVB irradiated for 6 h.

## Supplementary Materials

Supplementary materials can be accessed at: http://www.mdpi.com/1420-3049/21/10/1345/s1.
