# Supplementary material for: Ultraviolet B (UVB) Photosensitivities of Tea Catechins and the Relevant Chemical Conversions
Source: Molecules. 2016 Oct 10;21(10):1345. doi: 10.3390/molecules21101345 (PMC6274363; doi:10.3390/molecules21101345)
Supplement: Supplementary file 1 [file molecules-21-01345-s001.pdf]

Supplementary Materials: Ultraviolet B (UVB) Photosensitivities of Tea Catechins and the Relevant Chemical Conversions

Meng Shi, Ying Nie, Xin-Qiang Zheng, Jian-Liang Lu, Yue-Rong Liang and Jian-Hui Ye

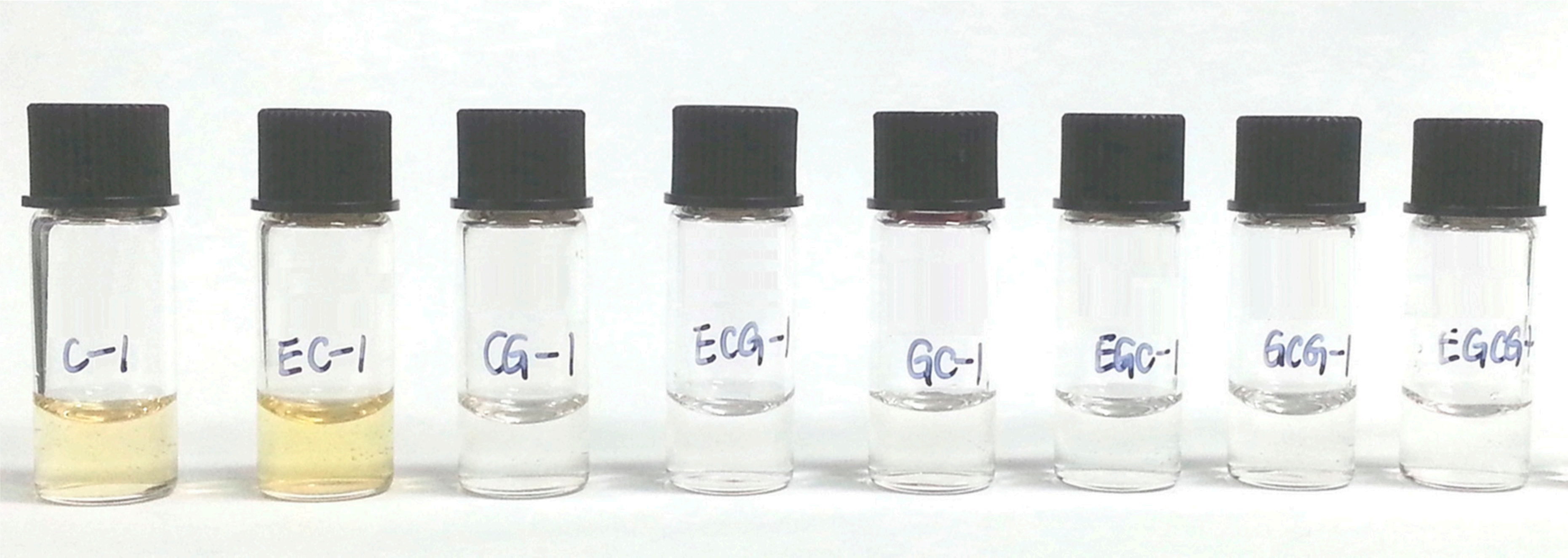

Figure S1. The picture of aqueous solutions of 8 catechins (575 μM) after 15-day-storage under laboratory illumination at 25 °C.
